# Reproductive decision making in women with medical comorbidities: a qualitative study

**DOI:** 10.1186/s12884-023-06093-4

**Published:** 2023-12-11

**Authors:** Elena M. Kraus, Niraj R. Chavan, Victoria Whelan, Jennifer Goldkamp, James M. DuBois

**Affiliations:** 1 Department of Obstetrics, Gynecology and Women’s Health, Maternal Fetal Medicine, Saint Louis University School of Medicine, St. Louis, MO 63117 USA; 2 Present Address: Department of Obstetrics & Gynecology, Creighton University School of Medicine, Omaha, NE 68178 USA; 3 Department of Obstetrics and Gynecology, Washington University School of Medicine, St. Louis, MO 63110 USA; 4 Mercy Clinic Maternal and Fetal Medicine, Saint Louis, MO 63141 USA; 5 Division of General Medical Sciences, Washington University School of Medicine, St. Louis, MO 63110 USA

**Keywords:** Qualitative research, High-risk pregnancy, Patient centered contraceptive counseling, Contraceptive access, Determinants of contraceptive use, Pregnancy planning, Unintended pregnancy

## Abstract

**Background:**

A growing number of reproductive-age women in the U.S. have chronic medical conditions, increasing their risk of perinatal morbidity and mortality. Still, they experience unintended pregnancies at similar rates to low-risk mothers. We have limited understanding of how these individuals consider decisions about pregnancy and contraceptive use. The purpose of this study was to understand factors that influence reproductive decision-making among pregnant women with chronic medical conditions.

**Methods:**

We conducted 28 semi-structured interviews with pregnant women with pre-existing medical conditions admitted to a tertiary maternal hospital to examine factors influencing reproductive decision making. Maternal demographic characteristics, medical history, and pregnancy outcome data were obtained through participant surveys and abstraction from electronic health records. Interview transcripts were coded and analyzed using Dedoose® with both deductive and inductive content analysis.

**Results:**

Out of 33 eligible participants, 30 consented to participate and 28 completed interviews. The majority of participants identified as black, Christian, made less than $23,000 yearly, and had a variety of preexisting medical conditions. Overarching themes included: 1) Perceived risks-benefits of pregnancy, 2) Perceived risks-benefits of birth control, 3) Determinants of contraceptive utilization, and 4) Perceived reproductive self-agency. Contraception was viewed as acceptable, but with concerning physical and psychological side effects. Although some considered pregnancy as a health threat, more experienced pregnancy as positive and empowering. Few planned their pregnancies.

**Conclusions:**

Preexisting health conditions did not significantly influence reproductive decision-making. Barriers to birth control use were generally based in patient value-systems instead of external factors. Interventions to improve uptake and use of birth control in this cohort should focus on improving care for chronic health conditions and influencing patient knowledge and attitudes toward contraception.

**Supplementary Information:**

The online version contains supplementary material available at 10.1186/s12884-023-06093-4.

## Background

Chronic medical conditions are increasingly common in reproductive age women^1^ in the United States (U.S), contributing to greater rates of high-risk pregnancies, severe maternal morbidity (SMM) and maternal mortality, particularly in minority populations [1–3]. Studies suggest that optimal use of family planning interventions, including contraception, could prevent up to 30% of maternal deaths worldwide [4]. Women with chronic diseases benefit significantly from planning pregnancy and have decreased eligibility for birth control options [4, 5]. The use of critical preventative services in this population, like preconception counseling, is overall low [6], and although these women plan for highly effective contraception, they experience unplanned pregnancies at a rate of 30–40%, similar to low-risk mothers [3, 7]. Women with comorbidities like hypertension, diabetes, epilepsy, and heart disease are less likely to receive contraceptive counseling, prescriptions, or services than those without these conditions [8–10], and postpartum contraceptive counseling following high-risk pregnancy is inconsistent [11].

It remains unknown why women with medical conditions, who arguably have a stronger rationale to prevent and plan pregnancy, are no more likely to use prescription contraceptives than those who do not. Limited qualitative investigation in this realm has revealed poorly understood discrepancies between medical needs, intention to use reproductive services, and actual use of these services [12–15]. Previously identified external influencers of such decision making include economic stability, education, community and social influences, legislation, and limits in insurance coverage [4, 16]. While social cognitive theory-based models have been used to explain contraceptive decision-making [15, 17, 18], few studies have explored how these decisions are made in real life through the lens of personal experiences. To this end, our primary research aim was to use qualitative methods to explore and describe factors influencing contraceptive decision-making and reproductive health behaviors among pregnant women with preexisting medical conditions.

## Methods

We used qualitative methods to gather and analyze data. Researchers working directly with participants had access due to being physicians in the hospital. All instruments were pilot tested with five antepartum and 5 postpartum inpatients through a structured cognitive interview process, and edited accordingly. A pre-interview survey and postpartum survey gathered demographic, pregnancy, and reproductive counseling information. Supplemental details of clinical history, outpatient care, and pregnancy outcome were acquired from a review of the electronic health records (EHR) by physicians and medical students with EHR access. Semi-structured interviews investigated participant views on pregnancy, motherhood, birth control, and influences of reproductive decision-making (Additional file 1: Interview Guide). We adopted the Theory of Planned Behavior (TPB) as a guiding framework for developing our interview guide and guiding our qualitative analyses [19]. According to this theory, a person’s intention and subsequent behavior is directly influenced by three main factors: beliefs and attitudes toward the behavior, personal subjective norms and the normative expectations of others, and the presence or absence of factors that facilitate or impede the behavior, also called perceived behavior control [20]. The interview guide was developed with several questions mapped to the TPB framework, as well as social-cognitive theory applied specifically to pregnancy planning and the use of contraception [17]. Within this framework, open-ended questions facilitated discussion of all potential contributing influences and considerations.

We approached pregnant patients admitted to the antepartum unit at an urban tertiary university-based teaching hospital. We recruited a purposive sample of participants at any gestational age, with English as their first language, and at least 16 years-old with medical conditions diagnosed prior to pregnancy. We considered any preexisting condition that increased an individual’s risk of experiencing pregnancy morbidity or mortality as defined by the Society of Maternal Fetal Medicine (SMFM) 2014 special report on the scope of Maternal Fetal Medicine (MFM) specialty services [1]. Eligible participants were informed about the study and invited to participate by the primary author and completed written informed consent prior to participation. The study protocol was approved by the university’s institutional review board (#31168).

Interviews were conducted in person in patient’s hospital rooms, by the primary author, who identified as a white woman and mother who was an MFM fellow, but who also had additional training and experience in qualitative methods through a PhD in Health Care Ethics. As such, some participants had met the researcher as a physician during outpatient prenatal care. Reflexivity was established through a post-interview journal where 8 standard questions addressed feelings, potential biases, contextual details, and other observations and non-verbal expressions noted in the interview. Interviews were audio-recorded, transcribed using standard language capture by a HIPAA-compliant web-based transcription platform, and de-identified interview transcripts were coded by 2 independent reviewers in addition to the primary author.

Coding was both deductive (guided by the TPB described above) and inductive insofar as themes within the TPB framework were not specified, and we were open to identifying themes that did not fit within the TPB [21]. Coding with conventional content analysis, as well as summative content analysis, was also performed [22]. Analysis was iterative, with data analyzed as interviews were completed and transcribed. Similar codes were combined into larger categories within the pre-existing frameworks of the TPB. Themes were developed to describe groups of codes and how codes related using the constant comparative method, and codes and themes were mapped to individual patient descriptors. Although thematic data saturation occurred at 15 interviews, due to the topic complexity and diversity of health conditions and lived experiences of participants, we recruited and interviewed until the max enrollment of 30 [23]. Dedoose® software was used for both interview and descriptor data management and analysis (9.0.18 ed). Consolidated Criteria for Reporting Qualitative Studies (COREQ) guided the research and result reporting [24].

## Results

**Fig. 1:**
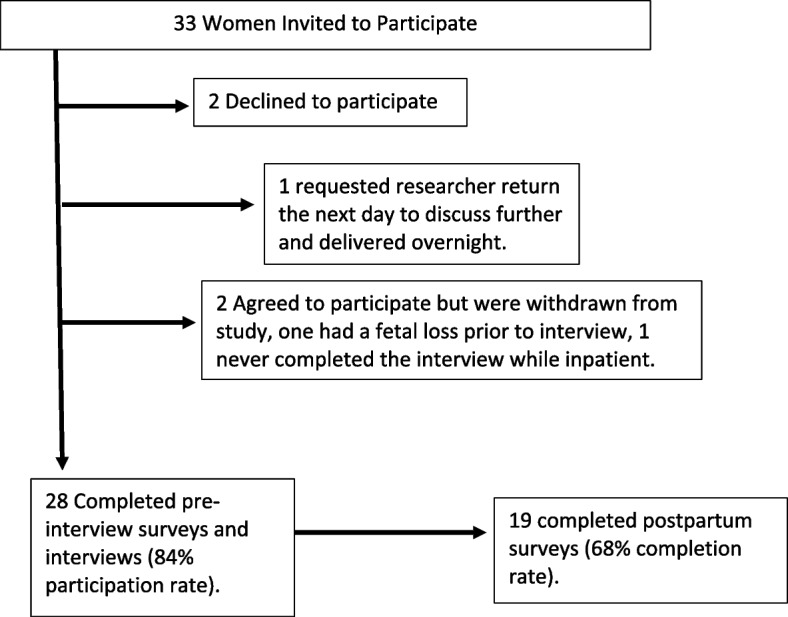
Participant recruitment for the study

Of the 33 pregnant women invited to participate, 30 enrolled, and 28 completed the pre-interview questionnaire and interview. Two participants were withdrawn prior to providing data; one never completed the interview prior to discharge, and another suffered a miscarriage prior to participation. Of those who completed the initial survey and interview, 19 completed the postpartum survey (Fig. 1: Participant Recruitment for the Study). Interviews ranged in length from 18 – 78 min. Average length was 31 min, 42 s. All participants received prenatal care, with 24 (86%) establishing care prior to 20 weeks. All had health insurance; 16 (57%) were supported by Medicaid or other public insurance. Most participants were interviewed in the third trimester (57%), made less than $23,000 per year (61%), were Black (64%), and multiparous (54%). Almost half (42%) reported using some type of birth control in the 6 months prior to pregnancy, and most (75%) had seen a non-obstetric physician within 6 months of their pregnancy. Participants had a variety of medical diagnoses and different intentions and birth control practices prior to pregnancy (Table 1).

**Table 1:** Characteristics of 28 participants

Characteristic	n (%)
**Age (years)**	
16–20	2 (7)
21–30	11 (39)
31–40	14(50)
41–45	1 (4)
**Race**	
Black or African American	18 (64)
White	8 (29)
None of the above	2 (7)
**Ethnicity**	
Hispanic or Latina	0 (0)
**Highest educational level**	
Less than high school	2 (7)
High School	8 (29)
Some college	10 (36)
College Degree	3 (11)
Graduate/Professional Degree	4 (14)
No response	1 (4)
**Employment status**	
Employed part-time	4 (14)
Employed full-time	11 (39)
Caregiver or homemaker	3 (11)
Self-employed	1 (4)
Unemployed	6 (21)
Disabled	2 (7)
No response	1 (4)
**Total yearly income before taxes**	
$0 – 23,000	17 (61)
$23,001 – 45,000	6 (21)
$45,001 – 75,000	2 (7)
$75,001 – 112,000	1 (4)
Greater than $112,000	2 (7)
**Religious preference**	
Christian	20 (71)
Catholic	1 (4)
Spiritual	2 (7)
None	2 (7)
None of the above	3 (11)
**Marital status**	
Married	11 (39)
Widowed	1 (4)
Divorced	0 (0)
Separated	3 (11)
Never been married	13 (46)
**Prior pregnancies**	
Nulliparous	13 (46)
Multiparous	12 (43)
Grand Multiparity	3 (11)
**Prior poor obstetric outcomes**	
Yes	9 (32)
No	19 (68)
**Considered high-risk in prior pregnancies?**	
I have never been pregnant before	7 (25)
Yes	16 (57)
No	5 (18)
**Maternal medical conditions prior to pregnancy**	
Substance Use Disorder	4 (14)
Mental health diagnosis	10 (36)
Type 1 or Type 2 Diabetes	9 (32)
Chronic Hypertension	13 (46)
Autoimmune diagnosis	4 (14)
Maternal Cardiac Disease	3 (11)
Asthma	10 (36)
HIV	1 (4)
Osteogenesis Imperfecta	1 (4)
**Saw a non-OB/GYN physician for a medical condition in 6 months before pregnancy?** (participant-reported)	
Yes	21 (75)
No	7 (25)
**Pregnancy desire**	
I had not thought about my desire to get pregnant	5 (18)
I did not want to get pregnant	4 (14)
I wanted to get pregnant	8 (29)
I wanted to get pregnant, but wished it was sooner	3 (11)
I wanted to get pregnant, but wished it was later	6 (21)
I felt unsure about getting pregnant	1 (4)
I did not want to get pregnant, but my partner wanted me to get pregnant	0 (0)
No response	1 (4)
**Pregnancy planning**	
I had not thought about planning for pregnancy	4 (14)
I did not plan this pregnancy	12 (43)
I planned this pregnancy	6 (21)
I was planning this pregnancy sooner	1 (4)
I was planning this pregnancy later	3 (11)
I did not plan this pregnancy, my partner planned this pregnancy	1 (4)
I’m not sure	1 (4)

Participant’s words illustrated a complex and value-based consideration of family planning and using birth control, with influences at multiple levels, from the individual person to the healthcare system, social influences, and faith. Primary themes and subthemes are presented in Table 2, with subthemes and representative quotes in Table 3. Subthemes mapped well to the primary components of the TPB. We combined our presentation of the TPB dimensions of “attitudes” and “norms” as these were frequently intertwined as participants discussed birth control, pregnancy, and influential factors in their considerations. Deliberations of becoming pregnant and using birth control involved attitudes, social norms, and considerations of self-efficacy, that ultimately led to intentions and behaviors. These considerations represented a risk–benefit comparison of both pregnancy and birth control use.

**Table 2:** Primary themes and subthemes

Theme	Subtheme
**Perceived Risks-Benefits of Pregnancy**	Desire for motherhood and children Pregnancy as empowering - Physical empowerment - Social Empowerment Pregnancy and birth experiences Influence of pregnancy on medical condition(s)
**Perceived Risks-Benefits of Birth Control**	Effects of birth control - Physical effects - Psychological effects Influence of contraception on medical condition(s) Reasons to use birth control
**Determinants of Contraceptive Utilization**	Partner influence Family support Social influences Physician counseling Birth control and healthcare access Financial stability Perceived susceptibility to pregnancy Perceived need for planning
**Perceived Reproductive Self-Agency**	Perceived control of pregnancy - Influence of self - Influence of higher power If it happens, it happens Birth control failure My body, my choice Birth control is easy to get

**Table 3:** Selected quotations illustrating subthemes

Subtheme	Representative Quote(s)
**Desire for motherhood and children**	The feeling of the baby move is beautiful. From the second that I felt him move, I felt more in love with him than I have anything [crying]. (Participant 16)
	I love children. I feel like children just put you in a happy space. I love being around them. I love molding them, and helping them grow, and just seeing their personalities. (Participant 13)
**Pregnancy as empowering**	I’m just strugglin’, but God has stepped in. Now I have a housin’ voucher. I was rentin’ right now, cash money. I have a voucher now. I’m tryin’ to find a house to move into. God has given me a chance to have a new baby. By me gettin’ pregnant is gonna change my whole life. (Participant 27)
	No. Everybody always said they don't think I will be able to carry kids because of the bone disease that I have and stuff like that, but I'm proving them wrong. (Participant 11)
**Pregnancy and birth experiences**	Because my last two pregnancies have been high-risk. I’m restricted on everything. I can’t do anything. I mean, for me personally, I don’t like to be pregnant. (Participant 3)
**Influence of pregnancy on medical conditions**	No, but I do feel like oftentimes I feel like people do take the whole high risk and pregnancy thing. When I first learned about it, it was really scary. Now that I’m so far along, and I’m getting ready to deliver, I feel like with the proper planning and with the proper medical attention, it’s definitely possible to have a, I guess, I wanna say like an ease of mind, like a better fit of mind going into a high-risk pregnancy. (Participant 24)
	As you know, diabetes and pregnancy is a full-time job. It went from being something that I fit into my life when I could, to becoming the main focus of everything that I did—every bite of food that I ate, every time my blood sugar was high, every time, like I said, just the constant anxiety of trying to do what was best. A lot of the fear that I had for the first trimester—holding my breath all the time wondering if I was gonna lose the baby. Especially with my first because I just figured, “I’m high risk. It’s not gonna work out the way I want it to. (Participant 22)
**Perceived susceptibility to pregnancy**	I just felt like it wasn't such a big deal just because it wasn't as easy for me to get pregnant, so I didn't have to force myself to get on [birth control] or anything like that. (Participant 21)
**Effects of birth control**	Then, I know when I had the Implanon, I—it was just spotting all the time, so I was like, "Nah, it's not worth it for me. (Participant 7)
	I noticed that it can help with mood and stuff like that, like changing people mood and that I’ve noticed being in the field I am and having improved some people moods and stuff being on birth control. I've seen that happen. I've seen it mess with their mood. Honestly, I think it shapes women different. I don't know. Their body shape, to me, I can tell, “Oh, she took birth control.” (Participant 30)
**Influence of contraception on medical conditions**	I guess it’s just about me having health conditions. It was rejectin’. It started to reject my liver. That’s why I feel the way I feel. (Participant 4)
	I knew after I got off and my blood pressure had started getting controlled, and it went down, so I—based on that, I just figured I was going to stop and not take any additional hormones or anything. (Participant 29)
**Reasons to use birth control**	I think just obviously my husband and I don’t want back-to-back children right away. Especially, revisiting it after this pregnancy would be something to discuss. (Participant 26)
**Partner Influence**	Really other than hubby being ready, no one really 'cause for me it's always been a very personal decision, and it's personal between the two people who are creating a baby. (Participant 7)
	There’s nothing that has really made me want to be pregnant. I mean, kind of—with this baby’s father, I wanted to share it with him. I wasn’t preventing, and it happened. (Participant 3)
**Family support**	I have my partner, and he was onboard. We were trying. Then you have my family who had actually even told him,’cause I did have a previous accident, and they told him, “Don’t get her pregnant. Don’t do it.” I’m thinking, how do you tell two adults who live in their own place [laughter] and who take care of things not to do something? (Participant 5)
	I know my mom changed a lot since she found out that I got pregnant. She's more excited than I am. It's basically like I'm carrying her child. (Participant 11)
**Social influences**	Then seeing all the family and the friends that I had around to help support me and all that actually helped me out a lot. Other than that, it wasn't such a big problem of being pregnant or anything. (Participant 21)
	Kinda sad because the people that I expected to be happy for me were not happy for me. I had more friends and colleagues. Well, colleagues were about 50–50 that were happy, and then they were like, “Well, why would you do that?” (Participant 5)
**Physician counseling**	Who would not really want to listen to their doctor, someone that’s paid millions of dollars for this education, works’round the clock nonstop? As humans, I feel like we always look for a go-to answer, whether it’s just us being lazy, or just because we don’t know. I think that the doctors play a huge part into it, but it’s still your choice. (Participant 5)
	Yeah. We had the thought, if we get pregnant, we get pregnant. If we don’t, we don’t. We talked to the doctors, who told me it should be a safe pregnancy. (Participant 29)
**Birth control and healthcare access**	Yeah. Any hospital I go into, I’ll be able to get on birth control if I wanted to or not—or my actual OB/GYN. If I go into his office and say, “Hey, Dr. so and so, I think I wanna go ahead and try this birth control right after I have my child,” they’ll be ready to give me the shot, give me the pill, give me the patch, give me the implant, give me whatever I need. I be able to get it. (Participant 28)
	It is, it’s offered—I mean, even at Planned Parenthood, it’s offered. I mean, there’s places where you don’t have insurance and they still help you. (Participant 3)
**Financial stability**	I'm not too concerned about financial. I'm not saying I make a lot of money, but I think I make enough to be able to take care of my baby. (Participant 30)
	My job and whether or not I had finances 'cause back when I was working jobs that I was making minimum wage, there's no way, no way. Now, being a little more financially stable, still not great but a little more financial stable, way more comfortable with the idea. (Participant 7)
**Perceived need for planning**	For me, it really just came down to feeling prepared, not that you can ever really be prepared, but feeling semi-prepared and not like I was totally just throwing a wrench into my whole life… (Participant 7)
	It really wasn't a thought, but I enjoyed the pregnancy and it wasn't a thought about, it wasn't planned or anything, but, yeah. (Participant 18)
**Perceived sense of control of pregnancy**	I think we all try to put this emphasis, “Well, it’s my body.” It’s the control. It’s control. It’s control. You’ve got women out there who want it more than anything, and they can’t do it. Then you have women who that’s the last thing they wanted on their mind, and now they have three or four kids. (Participant 5)
	It's everybody's own choice to use it or not to use it. I feel like when a baby is supposed to be here, they're gonna come whether you're on birth control or not. (Participant 12)
**If it happens, it happens**	I had regular menses that came every month after that, but you know you’ve gotta plan, and then you’ve gotta get the romance on. Then, at that time, I feel like my body was so stressed-out, and then I was getting sick all the time. I was getting low. I was getting low. I was like, there’s no way my body is going to be able to sustain a pregnancy right now, and lo and behold, I was wrong. (Participant 5)
	If it happens, it happens during whatever time. (Participant 30)
**Birth control Failure**	They did give me some pills without the hormones, without estrogen. That was pretty good. Then when I went to go get my tubes tied, I couldn’t get my tubes tied ‘cause I was pregnant. (Participant 19)
	Yeah, I was already—yeah, I got pregnant on birth control. Come to find out, my mother was, too, when she got pregnant with me. (Participant 16)
**My body, my choice**	Because you can pretty much control you. You just hear what other people have to say, but at the end of the day, you make your own decisions. (Participant 1)
	It’s my choice. If they refuse to wanna continue to treat me, I’ll go find another doctor. (Participant 28)
**Birth control is easy to get**	I think if you—it's fairly easy to use it once you get it. I feel like with all the different insurances and the different options, I feel like it's easy to get because now, I mean, you can order birth control on Facebook (Participant 12)
	I've never had any issues, because I've always had insurance. I've just always known where to go. I mean, you can go to the health department and get your sack of 50 condoms. (Participant 2)

### Attitudes and norms toward pregnancy and contraception

Attitudes toward pregnancy and contraception were intimately related. While participants frequently engaged in distinct risk–benefit analyses of each, attitudes toward pregnancy influenced attitudes toward contraception. Overall, participants’ opinions about pregnancy were far more positive than birth control. The general sentiments in their descriptions of pregnancy and motherhood were rich with emotions related to having children and expressed in terms of empowerment, a way to live out a life goal, and the increase in love that comes with having your own child.

“My general feelings about pregnancies are that it’s a wonderful thing. It may not be for everyone, but if it’s something you’re passionate about, I say, go for it. My feelings about pregnancies for me is that my kids are gifts.” (Participant 8)

On the other hand, general attitudes toward birth control were expressed with distrust and concern. Although most participants acknowledged its utility and had familiarity using a variety of methods, they described troubling experiences with contraceptives. Participants offered a wide array of physical side effects, from weight gain to abnormal bleeding, infertility, and feeling imbalanced or unnatural.

“Being on birth control myself, I tried it. It messes up my hormones and my pH balance. It wasn’t for me, but, I guess, it was the type of birth control. I was on the pill and the shot. I haven’t experience usin’ nothin’ else but them two.” (Participant 4)

The potential mental health effects of using birth control were mentioned almost as frequently as the physical side effects and led several participants to avoid contraception.

“I believe in birth control, I absolutely do. I believe that again is not for everyone. I had tried several different birth controls in the past, and they're not for me. Only because of my mental health issues. If I can get over my mental health issues then I'd be more willing to be on birth controls…” (Participant 8)

Participants’ own experiences with birth control and its effects on their health weighed heavily in their consideration for using it. It was repeatedly perceived as an intervention that could have negative health effects that lasted beyond discontinuation.

“I did try birth control one time, and it made my body just so wonky, and it was already wonky, so adding more chaos to it, I was just like, ‘Uh-uh. I can’t do that.’ I know a lot of people talk about, well, when they get off the birth control, it’s harder for them to conceive. It makes them feel really crappy. Personally, going through the not feeling good part, I was like, ‘I’m already sick enough. I can’t do this. No.” (Participant 5)

In contrast, pregnancy was noted to represent a temporary discomfort with the long-term gain of growing their family.

“I think that the pros as far as having my children outweighed the cons of the pregnancy. Being a mother and loving it outweigh the simple nine months that I spend creating the child and living in the constant anxiety. …I now see the sunshine at the end of the tunnel. It makes me a little bit more open to pregnancy. … It’s only temporary. Then, the baby will be here, and everything will be fine.” (Participant 22)

Another clear subtheme as participants weighed using birth control and pregnancy was health effects in relation to their specific medical conditions. Surprisingly, many participants described the interaction of birth control with their preexisting medical condition more readily than risks related to pregnancy.

“I have nothing against it, but I learned that birth control screwed up my blood pressure at one point. Yeah. We switched to condoms after that.” (Participant 29)

“I think a lot of people, including myself, have this really negative connotation attached in our brains when it comes to birth control. Especially with diabetes. If I’m vomiting, not feeling well, my blood sugars get high. There are estrogen pills that automatically make our blood sugars high. It’s just not always compatible, especially with high-risk bodies to be on birth control.” (Participant 22)

In contrast, less than half of participants explicitly related their medical conditions with risks in pregnancy. Most did not mention it until asked a specific question about how their health influenced these considerations.

“I think if I didn’t have diabetes, I’d probably—I don’t know ‘cause I’m kind of old. The age thing too. Yeah, that’s what I’m looking at age and health make me avoid possibly having more kids.” (Participant 30)

Participant narratives pointed to several factors that modified the perceived seriousness of their medical conditions in pregnancy. For many, the healthcare they received in the pregnancy mitigated poor outcomes, leading to the perception that pregnancy was not a significant threat.

“I’d do this all over again. Even though I’m high risk, I’d keep the baby. I’d continue my medical care. I’d definitely go through this all over again. I haven’t really had a bad experience even being high risk. I've been, of course, scared a lot of the times for my baby and for me, but I’d do this all over again. I feel like as long as you’re someone who’s going to keep up with your doctor’s visits and have a little faith and a little hope, then you can go ahead and carry on throughout with a happy pregnancy and a happy baby.” (Participant 24)

In many cases these perceptions remained despite counseling and awareness of the significant risks of pregnancy with their conditions. In these cases, their reassuring pregnancy experiences and outcomes positively influenced their views.

“I’m sure because I also have gotten lucky with the health of my other two children. That’s what influences me to be more open to pregnancy. Now, had the other two not gone as flawless as they did, then I may be quick to reconsider that. Considering being high risk, both my kids came out perfectly fine.” (Participant 22)

For others, interactions with the healthcare system enabled them to see how they could have a healthy pregnancy, despite their medical condition, and empowered them to be open to future pregnancies.

“Well, … at first was trying to avoid pregnancy because I was scared that ‘cause I have the HIV virus. I was scared and worried that my baby would come in contact with it. My doctors made me feel a lot more at ease knowing that there are medications that can help keep the baby from contracting the HIV.” (Participant 20)

Pregnancy was described by several participants as an experience that improved their health or the management of their condition, further promoting positive attitudes.

“I would be lying if I said I was not scared…but with the team that is here, I’m more than confident they can get me through it…Each time I’ve gotten pregnant and gotten this far, my sugars get better. The A1cs get better. I learn how to take care of myself better than what I did before, and just looking back on how much progress. You went from a person they said could not get an insulin pump, who’s constantly in DKA, who they said were gonna have amputated limbs, not have kids, all this type of stuff, to now having a pump…having my kids, and to have a baby that came out full term and perfectly healthy…Anything’s possible at that point.” (Participant 5)

Others expressed surprise at the complications they experienced and were more motivated to prevent pregnancy in the future as a result.

“I didn't know it would be this hard, this complicated. It’s stressful. It’s stressful and then making sure you basically manage your illness that you have and things like that. I think that would possibly be the main thing that makes me avoid having a kid.” (Participant 30)

Many other factors served as determinants of participants’ attitudes toward pregnancy and birth control. These included influences from partners, family, other relationships, physician counseling, and socioeconomic stability. There were examples of these determinants functioning both as a facilitators and a barriers to birth control use (Table 3).

Key additional contributors to participant attitudes and behavior were beliefs regarding infertility, and the perceived need for pregnancy planning. Eight participants expressed a belief they were infertile. Their reasoning was sound – many had unprotected intercourse for years and had not become pregnant, others were told by physicians that they were physically unable to get pregnant, and others held the misconception that their poor health meant their body could not support a pregnancy.

“Physically, I was told I was not physically able to get pregnant anymore. I was told that I was infertile. What was it they told me that—it would be a miracle if I did. They told me I would end up losing it before I was even six to eight weeks pregnant because of my cervix, so that doctor, wrong.” (Participant 16)

“I’ve had relationships I was in, and I tried. Then I didn’t get pregnant. That was in my teens and my early 20 s and stuff like that. Then I turned 32 and have a baby.” (Participant 17)

Their motivation for preventing pregnancy was reduced when the threat of pregnancy was not perceived as relevant. Still, some participants without prior pregnancies, and many with prior pregnancies with no reason to question their fertility, also did not see pregnancy as a salient threat. They approached the prospect of another pregnancy with ambivalence about needing to prevent it. Accordingly, most participants did not express preemptive planning of pregnancy, even in the context of their health conditions. Without prioritization of family planning, there was reduced motivation to seek out birth control.

“We never decided to have—it just came. We never said ‘Okay, we gonna—’ we never said that. We can’t even afford a damn strawberry daiquiri. Ain’t gonna sit here and say, ‘Okay, we gonna have this baby.’ We never planned it out. … It just came.” (Participant 27)

In contrast, a handful of participants with careers, educational programs, and other competing responsibilities described in detail how they had planned for the current pregnancy.

“We made a checklist of stuff we wanted to do before pregnancy, and we hit most of them. COVID did throw some stuff off, but we got a lot of our traveling in that we wanted to. Started feeling more secure in my career. Made it open for us to do it then ‘cause we thought it’s the right time.” (Participant 29)

Participants’ motivation for contraceptive use or non-use was not based solely on attitudes, norms, and other determinants of birth control. Competing with these internal and external factors were a similar and parallel consideration of pregnancy, which brought the benefits of love, empowerment, and growth of relationships or family. Many perceived the risks of using birth control to be more salient than the risks of pregnancy. In particular, the perception of obstetric risk was diminished for many by their engagement with the health system and prior good experiences and outcomes. Another clear modifier was the participants’ belief in their ability to conceive. Participant comments exhibited an explicit weighing of the perceived risks and benefits of pregnancy as well as using contraception.

“I think you got your good and your—I mean, you got your pros and your cons with everything, but it’s more like the risk and benefit. Like, are you willing to risk this or to not have that?” (Participant 19)

### Perceived behavioral control over reproduction

Participants’ expression of how they controlled their reproductive options illustrated both an expressed power over their reproductive choices, as well as a concession that it was not entirely in their control. All participants uniformly expressed a perceived ability to acquire and use contraception, which exerted some control over pregnancy. Only three participants described experiences with barriers to obtaining birth control, which they all overcame by using an alternate method.

“I think it's very easy. I really think you have––well, we have access to getting birth control. Even just going online, yeah, it's very easy.” (Participant 30)

Several others expressed a similar control over pregnancy in decisions about who they had sex with and whether they chose to use birth control.

“I have control over if I want to sleep with you. Do we want to use a condom, am I on birth control.” (Participant 18)

Although there were many social, familial, and medical influences on their individual decisions about family planning, almost all participants strongly expressed that regardless of what other people thought, they could and would make their own decisions regarding reproduction.

“After I have this baby, I don’t wanna get pregnant again. I’m not gonna be stingy. I’m satisfied. I’m happy with the babies that I have. I also don’t think it’s their right to tell me if I should be able—if I have more children or not, if I should be still fertile, or I shouldn’t because that’s my choice. My body, my right.” (Participant 16)

Despite these expressions of control over contraceptive use and intimacy, most also expressed a strong sense that a greater power was at work with pregnancy, and that the ability to get pregnant was not completely under their control. Many referenced issues with infertility or pregnancy loss and articulated there were no guarantees in achieving or avoiding pregnancy.

“I mean, I can only do so much about birth control or stopping birth control, but God is in control,’cause even when we first started trying to conceive, I stopped birth control, but I had been on birth control for six years, so of course it took a while’cause the stuff was still in my system. Then having a miscarriage, it’s just like, “Okay, maybe it’s’cause I was on birth control. Somehow my body couldn’t regulate yet or something.” You can only do your part, and it’s not 100 percent guarantee that you’re gonna get pregnant or that it’ll fertilize or whatever.” (Participant 23)

Faith in a higher power influenced their belief that having a baby was not entirely in their control, and helped them understand and accept related outcomes.

“I don't think so. I think it's up to God. I really do ‘cause ain’t no way––yeah, ‘cause even though I use the condom method, it wasn't used a lot, but then we almost 10 years down the road and I get pregnant. I really think it's up to God.” (Participant 30)

Participants expressed strong feelings of autonomy, and at the same time, a vulnerability and almost helplessness to change what is dictated by this higher power. Interestingly, participants did not express distress at this lack of control The subtheme “if it happens, it happens,” was expressed repeatedly, revealing an ambivalence about becoming pregnant, and an acceptance of whatever happened. This sentiment seemed driven by the positive emotions most participants associated with having children, and a lack of willingness to change course once pregnancy was established.

“Doesn't bother me. Not one bit. Because at the end of the day, I know what I want in life, and I'm gonna get it, regardless…I believe in God to the fullest extent, and I believe in letting it—whatever's supposed to happen for you is going to happen, regardless. There's nothing you're gonna be able to do to stop it, to change it. It's gonna happen, you know, so, I mean, I don't plan on doin' any more of this, but if I were to come up with another kid, he or she would be well taken care of just as well as this one.” (Participant 25)

“Um, nothing really influenced me to get pregnant, but like—'cause it technically just happened. It was just really take or leave it 'cause it already had happened, so it was like if anything bad happens, there's nothing really you can do. Just live with it.” (Participant 1)

Five participants described birth control failure, further propagating the perception that absolute control was not possible, even when contraceptives were used.

“I got pregnant with my twins—I got pregnant with the twins when I had the Implanon.” (Participant 19)

Overall, participants expressed both strong decision-making power and ability to access contraception if desired, but an underlying understanding that pregnancy is something you cannot fully control.

“You are the master of your ship, but you’re not the master of the seas.” (Participant 5)

## Discussion

This study contributes substantive insight into the reproductive health behaviors of women with medical comorbidities by investigating women’s views of both pregnancy and contraception. Our data suggest that in a predominantly lower income, black pregnant population receiving healthcare, reproductive decision-making is most significantly influenced by individual value systems, and often without consideration of the impact of health conditions. Pregnancy was generally viewed positively and worthwhile despite discomforts and complications. Although many were counseled and knowledgeable about the risks of pregnancy related to their health, their understanding of pregnancy as a threat was modified by beliefs that risks were theoretical or transient, and good outcomes with health engagement in pregnancy. Birth control was considered necessary and useful, but had risks and side effects as salient as those of pregnancy. Taken together, these results help explain data indicating women with preexisting medical conditions neither use birth control more frequently nor have fewer unintended pregnancies than healthy women [3, 8, 10].

Participants expressed a high degree of perceived control over birth control access and use, and negative attitudes about contraception, which has been described in other studies [25–27]. Perceived behavioral control correlates with birth control behavior [25]. However, our results suggest that concomitant views of pregnancy in the context of relationships and family growth modify this sense of self-agency, decreasing the motivation to use contraception. These results echo prior qualitative studies showing ambivalent, fluctuating, and incongruous feelings toward pregnancy and birth control and distrust of hormonal contraception, all strongly influenced by personal and second-hand experience [14, 15, 28, 29]. A far too recent history of forced sterilization and reproductive coercion in healthcare may also contribute to negative attitudes, particularly among marginalized populations [30]. Ambivalent attitudes toward contraception are associated with decreased contraceptive use and increased unintended pregnancy [31].

Using the TPB framework helped categorize and interpret participants’ perspectives, and highlighted key attitudes and beliefs our participants considered when making reproductive decisions. Our findings paralleled theoretical work using social cognitive models to understand contraceptive decision-making [17, 18], but also highlighted the shortcomings of the TPB applied to family planning and contraception. First, our participants generally did not plan pregnancy, nor describe significant rational engagement with the risks of pregnancy in their reproductive decision-making and behavior. Unlike many diagnoses, pregnancy was not perceived by our participants as a threatening health condition, but rather a means to family growth with strong positive emotional sentiments. Second, contraception use is a unique health behavior in that it does not necessarily treat pathology or symptoms, but for many of our participants, created new symptoms. With no perceived improvement in health and the burdens of use, women lack motivation for initiation and are not reinforced in long-term adherence. These results provide insight into the common paradox of patients who express a desire to avoid pregnancy and deny barriers to obtaining contraception, but who do not ultimately use contraception.

It is important to use theoretical models to design and implement effective interventions that target specific mechanisms of behavior to change outcomes [32]. This study points to individual values and norms as the most influential factors impacting women’s motivations to prevent pregnancy. Our findings help explain why prior interventions based on external barriers have succeeded in increasing availability and reducing cost, but have not significantly changed outcomes [33, 34]. Interventions to increase uptake and continued use of birth control would benefit from a focus on understanding and shaping patient value systems through reflective listening and counseling, education, and influence of community opinion leaders. Another key approach to education and influencing attitudes is a focus on increasing access to high-quality primary care for reproductive-age women with medical conditions. In the state where this study was performed, health insurance is provided for all pregnant women, but not guaranteed outside of pregnancy. Optimizing pre-pregnancy healthcare engagement would likely improve overall health as well as increase motivation for intentional reproductive choices. Targeted interventions have the potential to improve reproductive planning and ultimately decrease maternal morbidity and mortality by avoiding or delaying pregnancy in women at increased risk for adverse maternal and fetal outcomes [4].

This study has limitations. Our sample was predominantly black and insured; it is unclear whether studies with different populations would uncover similar decision-making dynamics. Our participants were all pregnant and chose to continue pregnancy despite their medical conditions and complications that developed necessitating hospital admission; thus, they may have different attitudes about pregnancy and less positive views about birth control than non-pregnant women with medical conditions. Participants were all receiving high-quality healthcare in a religiously-affiliated healthcare system, which similarly focuses on a population needing healthcare and receiving a high level of health services. Our participants represent an important demographic served by women’s health specialists.

## Conclusions

Our findings challenge assumptions and improve our understanding of why women with chronic medical conditions do not use contraception at higher rates than other women, and demonstrate the value and limitations of frameworks such as TPB for understanding the reproductive choices of women. These results underscore the multifaceted consideration of contraception and pregnancy undertaken by women with medical conditions, and suggest ongoing research should address the complex interplay of subconscious and conscious processes when attempting to understand or address women’s reproductive behaviors. This research contributes to an evidence-base that can help guide interventions that have the potential to change reproductive decision-making and behavior and improve maternal outcomes.

### Supplementary Information

**Additional file 1.**

## Data Availability

The datasets used and/or analyzed during the current study are available from the corresponding author upon reasonable request.

## Abbreviations

U.S: United States
SMM: Severe Maternal Morbidity
COREQ: Consolidated Criteria for Reporting Qualitative Studies
TPB: Theory of Planned Behavior
SMFM: Society for Maternal Fetal Medicine

## Footnotes

1 The Society of Maternal Fetal Medicine (SMFM) has adopted the use of the word “woman” (and the pronouns “she” and “her”) to apply to individuals who are assigned female sex at birth, including individuals who identify as men as well as nonbinary individuals who identify as both genders or neither gender [4]. This manuscript uses the term in this way while also using gender-neutral language where possible.
