# Supplementary material for: Reproductive decision making in women with medical comorbidities: a qualitative study
Source: BMC Pregnancy Childbirth. 2023 Dec 11;23:848. doi: 10.1186/s12884-023-06093-4 (PMC10712035; doi:10.1186/s12884-023-06093-4)
Supplement: Supplementary file 1 — Additional file 1. [file 12884_2023_6093_MOESM1_ESM.docx]

**Additional File 1: Interview Guide**

1. What are your general feelings about pregnancy? What are the main factors that influence these feelings?
2. What are your general feelings about motherhood? What are the main factors that influence these feelings?
3. What are your general feelings about using contraception, or birth control? What are the main factors that influence these feelings?
4. What personal characteristics or experiences are the biggest influencers on your choice to be open to pregnancy or avoid pregnancy?
5. What changes did you experience in your life when you became pregnant?
6. What sources of information do you value the most when considering what birth control to use, or whether to use birth control at all?
7. How have other people in your life influenced your choice to get pregnant or avoid pregnancy? What do you think are other peoples’ expectations of you regarding birth control?
8. What other things in your life have influenced your choice to get pregnant or avoid pregnancy?
9. How has your health influenced your choices in getting pregnant or avoiding pregnancy?
10. How have the experiences of your current or past pregnancies influenced your choices in getting pregnant or avoiding pregnancy?
11. Do you think the beliefs of your medical providers affect your options for birth control? If so, how?
12. Do you think the religious affiliation of the healthcare institution you are using affects your options for birth control? If so, how?
13. Do you feel you have control (or choice) over when you get pregnant? Why or why not?
14. How easy or difficult do you think it is to get and use contraception or birth control? What makes it easy or difficult? Were you ever unable to get something you wanted?
15. What do you want to hear from your physicians about contraception or birth control? When do you think they should discuss it with you?
16. Describe your ideal contraceptive or birth control method.
17. How did COVID-19 affect your pregnancy? Did your views on pregnancy or getting pregnant change as a result of the COVID-19 pandemic? How did they change?
